# Local peritonitis and omental bursitis as sequelae of abomasal ulceration in buffaloes (*Bubalus bubalis*): clinical findings and outcome of treatment

**DOI:** 10.1080/01652176.2020.1718796

**Published:** 2020-01-30

**Authors:** Shaimaa M. Gouda, Ahmed M. Abdelaal, Shimaa A. Elgaml, Shimaa A. Ezzeldein, Eslam F. Eisa, Emad A. Hashish

**Affiliations:** a Department of Animal Medicine, Faculty of Veterinary Medicine, Zagazig University, Zagazig, Egypt; b Department of Clinical Pathology, Faculty of Veterinary Medicine, Zagazig University, Zagazig, Egypt; c Department of Surgery, Anesthesiology and Radiology, Faculty of Veterinary Medicine, Zagazig University, Zagazig, Egypt

**Keywords:** Buffalo, abomasal ulcer, omental bursa, bursitis, peritonitis, ultrasonography

## Abstract

Abomasal ulcers are difficult to diagnose clinically with limited therapeutic approach to combat the disease. Omental bursitis (OB) and local peritonitis (LP) are known sequelae of abomasal ulcer in cows. In this study, differentiation between OB and LP in regard to clinical symptoms, biochemical analyses and the response to treatment in Buffaloes was done. Twenty buffaloes (*Bubalus bubalis*) were admitted with a history of intermittent appetite, wasting and mild abdominal distension during the period between March 2016 and August 2018. All cases were female (12 recently calved, 2 pregnant, 6 non-pregnant) aging from 3 to 9 years and weighing 350–600 kg. For comparison, 10 apparently healthy non-pregnant female buffaloes were used as controls. Abdominal ultrasonography confirmed the presence of 11 OB and 9 LP in admitted cases. Laboratory analysis revealed hyperproteinemia and hypoalbuminemia in OB. Hypokalemia and hypochloremia were detected in both OB and LP. Ultrasonography showed hypo-anechoic content with echoic stippling surrounded by echogenic wall in OB, whereas echogenic strands interspersed with anechoic fluid was reported in LP. Intra-lesional lavage by normal saline was applied several times under ultrasongraphic guidance followed by gentamicin 10% intramuscular and H2 antagonist intravenous for 5 days as well as parenteral and enteral fluid therapy. Seven cases of OB clinically improved, whereas no improvements were found in LP cases. OB secondary to abomasal ulcer has a good prognosis in contrast to LP. Ultrasonography provides a useful diagnostic tool and therapeutic guidance for such diseases.

## 1. Introduction

Abomasal ulcer is a condition which commonly occurs in cattle. Its severity varies from mucosal erosion to perforation of abomasum with a rapid development of peritonitis. Several factors like stress, the use of anti-inflammatory drugs, periparturient diseases and moist high acidic rations either concentrates and/or silage have been considered as most important regarding abomasal ulcer induction in dairy cattle. Perforation of abomasum might subsequently be accompanied by local, diffuse peritonitis and/or omental bursitis (Constable et al. 2017).

Omental bursitis in cattle is a rare inflammatory condition of the omental bursa (Cavum bursa omentalis). Recently, it was defined as a special form of peritonitis when limited to the bursa and demarcated by a capsule from the remaining abdominal cavity (Braun 2016). As such it is regarded as a sequelae of abomasal ulcer especially when the medial wall of the abomasum is involved. In addition, it might be induced due to foreign bodies penetrating the ventral wall of rumen or reticulum or secondary to perforation of small intestine or laparotomy (Peek and McGuirk 2008). The spillage of ingesta into this bursa might lead to its inflammation and subsequently stimulates the formation of inflammatory exudate (Constable et al. 2017).

Clinically, omental bursitis is difficult to diagnose as the patients either young (Sardari 2007) or adult cattle (Fubini and Ducharme 2016) are presented with nonspecific signs including anorexia, abdominal distension, chronic toxemia and dehydration.

Although ultrasonography has been widely used as a diagnostic and prognostic tool in the bovine species with abdominal disorders, there is limited ultrasonographic data describing omental bursitis in cattle and no available data exist about its occurrence in buffaloes. For all of these, the present study was undertaken to investigate the clinical, biochemical analyses and therapeutic response of buffaloes with omental bursitis compared with other cases that have local peritonitis secondary to abomasal ulcers as well as throw the light on the utility of ultrasound in such diseases.

## 2. Materials and methods

### 2.1. Animals

Twenty buffaloes (*Bubalus bubalis*) were admitted to Veterinary Teaching Hospital, Zagazig University, Egypt during the period between March 2016 and August 2018. All cases were female (12 recently calved, 2 heavily pregnant, 6 non-pregnant), aged from three to nine years and weighed from 350 to 600 kg. For comparison, 10 apparently healthy non-pregnant female buffaloes obtained from the farm belonging to Zagazig University were used as controls. The affected animals were admitted by a history of intermittent appetite, wasting and mild abdominal distension. All affected animals were previously treated by intravenous saline, lactated ringer, antibiotic, purgatives and rumenotonics by referring veterinarians. Complication of perforated abomasal ulcer was tentatively diagnosed on the basis of history, clinical findings, and the results of laboratory investigations, while confirmation was achieved by abdominal ultrasonographic scanning or by necropsy (*n* = 13). Based on ultrasonography, the diseased buffaloes were categorized into abomasal ulcer with omental bursitis (OB; *n* = 11) or local peritonitis (LP; *n* = 9).

The care and welfare of the animals were conformed to the guidelines of the Animal Use Research Ethics Committee, Zagazig University, Egypt.

### 2.2. Clinical examination

Vital parameters including rectal temperature as well as heart and respiratory rate were reported for both control and diseased animals. Each animal was assessed for general condition, abdominal contour, abdominal pain reactions, defecation, urination, ruminal contractions, simultaneous percussion and auscultation of the abdomen as described previously (Rosenberger 1990).

### 2.3. Ultrasonography and ultrasonographic guided abdomino-centesis

Abdominal scanning was applied by ultrasound adopted from the protocol previously described (Braun 2009). Firstly, the area of ventral abdomen caudal to the xiphoid till the umbilical region (cranio-caudal), then both lateral sides of the abdomen were scanned by ventro-dorsal technique (Sonoscape, A5V, Guangdong, China) with 3.5 and 5 convex transducers. Whole area was first clipped and coupled by coupling gel (Aquasonic100, Parker laboratories, Fairfield, NJ, USA). Ventral abdominal scanning was applied for evaluation of the reticulum and abomasum, left lateral scanning for evaluation of the rumen, reticulum and spleen while right lateral scanning was applied for evaluation of the reticulum, abomasum, omasum, intestine and the liver.

Abdomino-centesis was applied by directing the needle (10 cm length, 14 gauge) towards the detected lesion under ultrasonographic guidance after disinfection of the entry seats using alcohol 70%. Physical characters included color, odor, consistency and quantity.

### 2.4. Blood sampling

Ten milliliters blood samples were freshly collected from the jugular vein of all diseased and clinically healthy buffaloes for analyses of the serum biochemistry. Blood which flow smoothly into tubes without anticoagulant were used for the serum separation and biochemical analysis.

### 2.5. Laboratory examination

#### 2.5.1. Biochemical parameters analysis

All parameters were colorimetrically estimated by using commercial kits (BioMérieux, Marcy-L’Etoile, France). All the analyses were done by spectrophotometry (Spectrophotometer 5010 v5+, RIELE GmbH & Co, Berlin, Germany) for estimation of the serum biochemical parameters. The concentration of serum total protein and albumin were estimated according to previous publications (Doumas et al. 1972; Weissman et al. 1974, respectively). Meanwhile, the concentration of serum globulin was calculated by subtracting the concentration of obtained albumin from the concentration of obtained total protein. The concentration of serum pepsinogen was determined as previously described (Khalphallah et al. 2018). Serum sodium (Na), potassium (K) and chloride concentrations were determined by using a flame photometer (Jenway, PFP7 Clinical, Essex, England) (Verzhikovskaia and Popov 1963).

### 2.6. Treatment procedure

The treatment procedure was applied as follows: drainage of the obtained fluids via aseptic aspiration, intra-lesional lavage with saline 0.9% (Misr Co, Al-qalybia, Egypt) followed by systemic antibiotic, H2 antagonists and fluid therapy regarding both OB and LP. The selected antibiotic was Gentamicin 10% (Gentacure^®^; Pharma Swede, Pharmaceutical Co, 10th Ramadan, Egypt) at a dose of 4 mg/kg BW intramuscularly daily for 5 days (Boueil et al. 2015). Ranitidine (Rani^®^ Ampoule, Pharco Pharmaceutical Co, Cairo, Egypt) at a dose of 1 mg/kg BW intravenously bid for 5 days was the H2 antagonists used in this investigation. Fluid therapy (10 L/head/day) either saline or Ringer’s solution (Misr Co, 10th Ramadan, Egypt) parentally and orally (500 g magnesium oxide, 100 g sodium chloride and 50 g potassium chloride/20 L water/head) were used daily upon the course of treatment (Constable et al. 2017). Follow-up information was collected through owner contact and re-evaluation was applied after 7 days.

### 2.7. Necropsy findings

Necropsy findings including origin and extensions of the lesion were applied on 13 buffaloes that did not respond to the treatment.

### 2.8. Statistical analysis

Statistical Package for Social Sciences (SPSS) version 17.0 (IBM, Chicago, USA) was used. Parametric analysis of variance (general linear model) was used for one-way ANOVA test. The differences between groups were compared by using post-hoc Bonferroni multiple comparison tests. *P* ≤ 0.05 was considered statistically significant.

## 3. Results

### 3.1. Clinical findings

The result of clinical findings is summarized in Table 1. Out of the 20 studied diseased buffaloes, 11 were affected with OB and 9 animals showed LP. Both groups demonstrated common clinical findings including systemic disturbances with congested visible mucous membrane, altered appetite either decreased or ceased with decreased ruminal motility or ruminal stasis and varying degrees of dehydration. Abdominal distension was visible at right ventral abdomen in 4 cases of OB and in 7 cases of LP cases. Expiratory grunt was noted in 3 cases of OB and 4 cases with LP. Fluid splashing sound was evident among 4 cases of OB and 4 cases of LP. The majority of OB cases (*n* = 9) and LP (*n* = 6) had scanty feces and fewer had diarrhea. Melena was detected in 6 cases of OB and 4 cases of LP. Dullness and depression with a state of unconsciousness were observed in 4 cases of OB and in all LP cases. Recumbency was an additional uncommon sign registered in 3 cases of LP cases.

**Table 1.** Frequency distribution of clinical findings in diseased buffaloes (OB and LP) cases in comparison with control.

	Control ( *n* = 10)	OB ( *n* = 11)	LP ( *n* = 9)
Systemic reactions^a^	0	8	9
Congested conjunctiva mucous membrane	0	8	9
No dehydration	10	0	0
Mild to moderate	0	11	0
Severe	0	0	9
Good appetite	10	0	0
Decreased	0	7	0
Anorexic	0	4	9
Visible abdominal distension^b^	0	4	7
Expiratory grunting sound	0	3	4
Ruminal contractions/auscultation			
Normal (3–5/2 min)	10	0	0
Decreased	0	8	0
Absent	0	3	9
Splashing sound by abdominal ballottement	0	4	4
Normal defecation	10	0	0
Scanty feces	0	9	6
Diarrhea	0	2	3
Melena	0	6	4
Unconsciousness	0	4	9
Recumbency	0	0	3

^a^ Systemic reactions (elevated body temperature above 39 °C, heart rate above 80, and respiratory rate above 30).

^b^ Visible abdominal distension was at right ventral abdomen.

### 3.2. Ultrasonographic findings

**Figure 1.**
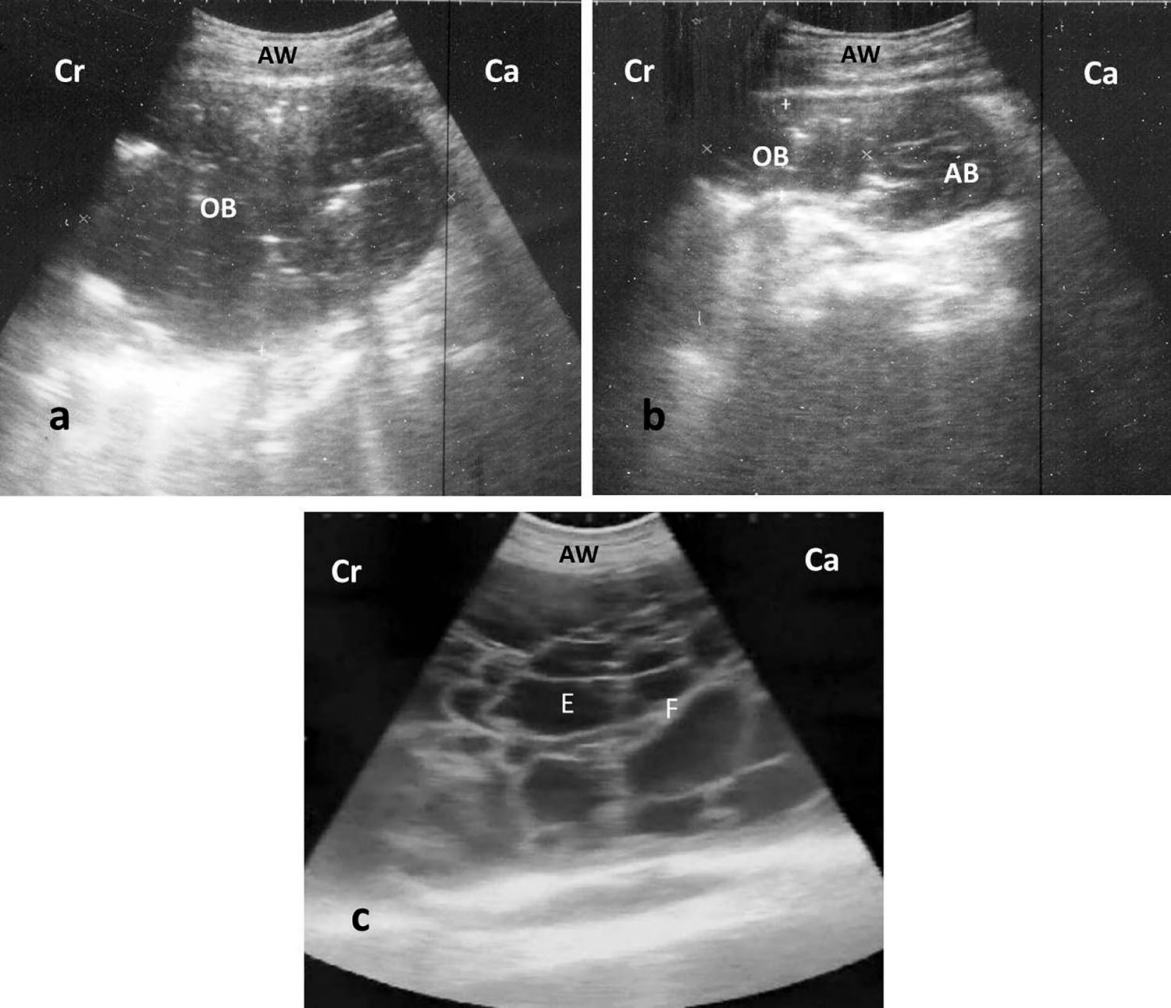
Ultrasonography of ventral abdomen with 3.5 MHz convex probe show: (a) anechoic content with echoic stippling and surrounded by echogenic wall (OB), (b) same structure after aspiration; notice the appearance of abomasum (AB) after reduction of bursal size, (c) peritonitis appears as echogenic brands (F: fibrin) interspersed with anechoic fluid (E: exudate). AW: abdominal wall, Cr: cranial, Ca: caudal.

Abdominal ultrasonographic scanning of all affected buffaloes (*n* = 20) revealed normal reticular shape with decreases in contractions (1–3/5 min) compared with control (4–6/5 min). Enlarged omental sac with echogenic wall and 10–15 cm in size was shown in 11 affected buffaloes and its content varied between anechoic to slight hypoechoic with echoic stippling (Figure 1(a)). Aspiration of the enlarged bursa made the abomasum visible (Figure 1(b)). Peritonitis was observed as echogenic debris or strands interspersed with anechoic fluid in 9 cases (Figure 1(c)). All lesions either OB or LP were observed at ventral abdomen.

Re-evaluation of OB cases by ultrasonography after one week revealed a decrease in the size of bursitis (*n* = 4), disappearance of bursitis with visible abomasum (*n* = 3) or peritonitis as a sequelae (*n* = 4). In contrast, LP cases showed no response at all and the peritonitis became more diffuse in all cases. Hence these animals were slaughtered given the poor prognosis.

The aspired fluid was creamy yellow with foul odor and copious amount in OB while it was scanty, watery and foul smelling in LP cases.

### 3.3. Laboratory findings

#### 3.3.1. Biochemical changes

##### 3.3.1.1. Changes in proteinogram

The OB cases showed alterations in the protein spectrum as manifested by a significant (*P* ≤ 0.05) increase in the serum total protein and globulin concentrations with a significant decrease in the serum albumin concentration when compared with the controls. LP cases revealed a significant (*P* ≤ 0.05) increase in the serum globulin concentration in comparison to the control cases. All the changes in the protein spectrum in OB and LP cases are listed in Table 2.

**Table 2.** Biochemical findings in buffaloes with omental bursitis (OB) and local peritonitis (LP) secondary to abomasal ulcer in comparison with the control.

	Control (*n* = 10)	OB (*n* = 11)	LP (*n* = 9)
Total protein (g/L)	70.5 ± 2.0 b	80.7 ± 3.0 a	80.0 ± 2.0 ab
Albumin (g/L)	42.2 ± 1.0 a	37.4 ± 1.0 b	45.0 ± 1.0 a
Globulin (g/L)	28.3 ± 1.0 b	43.3 ± 2.0 a	35.0 ± 1.0 a
Pepsinogen (units/L)	3.85 ± 0.08 b	9.3 ± 0.57 a	10.0 ± 0.5 a
Na (mmol/L)	140.3 ± 0.07 ab	135.2 ± 2.99 ab	134.33 ± 3.5 b
K (mmol/L)	4.9 ± 0.07 a	3.7 ± 0.3 b	4.06 ± 0.34 b
Cl (mmol/L)	102.5 ± 0.95 a	96.2 ± 3.4 b	86.3 ± 0.3 c

All data that have different letters are significantly differed at *P* ≤ 0.05.

##### 3.3.1.2. Changes in pepsinogen concentration

The concentrations of pepsinogen in buffaloes affected with OB and LP are listed in Table 2. Results showed a significant (*P* ≤ 0.05) increase of the pepsinogen concentration in OB and LP cases when compared with the control cases.

##### 3.3.1.3. Changes in sodium, potassium and chloride concentrations

Alterations in the electrolyte concentrations were detected and manifested by significant (*P* ≤ 0.05) decrease in the serum potassium and chloride concentrations in all buffaloes suffering from OB and LP comparing with the control cases. Changes in the serum sodium, potassium and chloride concentrations are listed in Table 2.

### 3.4. Treatment procedure and necropsy findings

Out of 11 OB cases, 7 cases responded to treatment and showed improvement in clinical signs while the other cases got worse and became complicated by peritonitis and paralytic ileus. On the other hand, all 9 cases of LP did not respond to treatment.

**Figure 2.**
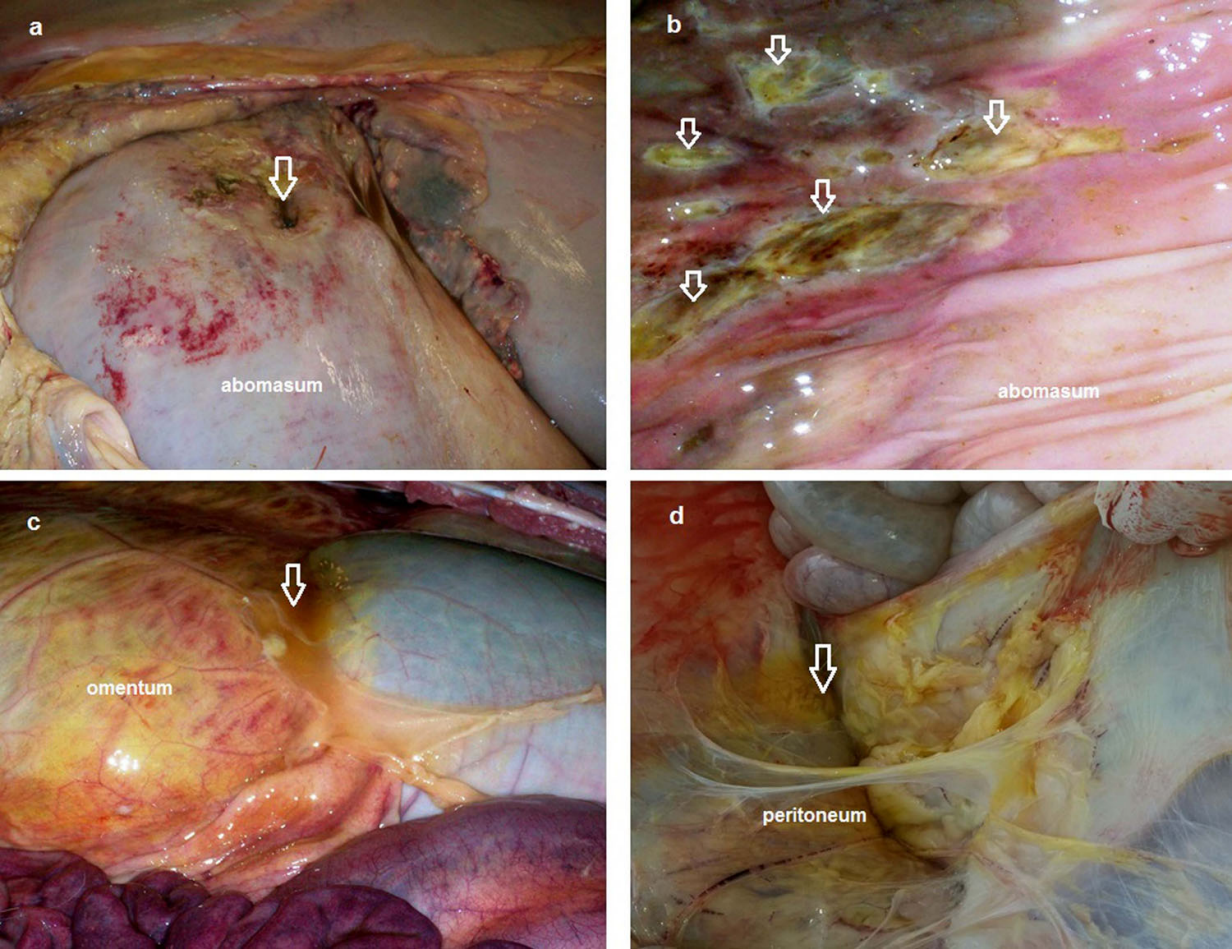
Necropsy findings show: (a) single perforated abomasal ulcer appears from the outer wall (arrow), (b) multiple non-perforated ulcers appear from inner wall (arrows), (c) ruptured omental bursa with peritonitis (arrow) and (d) diffuse peritonitis with adhesion of visceral organs (arrow).

The 13 cases that not responded to treatment were slaughtered or necropsied. These 13 cases showed round perforated ulcers with large size (2–5 cm in diameter) observed at the greater curvature of abomasum. Blood and necrotic material were observed at the site of the ulcers (Figure 2(a)). Other multiple non-perforated ulcers were observed at the inner mucosa (Figure 2(b)). Rupture of omental bursa with peritonitis was observed in 4 cases (Figure 2(c)), whereas adhesions between perforated ulcer and omentum, peritoneum and intestine were observed in all 13 cases (Figure 2(d)).

## 4. Discussion

Perforating abomasal ulcers are considered as the most common cause of omental bursitis and peritonitis. Diffuse peritonitis in ruminants is regarded as a potential fatal inflammation as well as the most common cause of peritonitis-related death worldwide (Elgioushy et al. 2019).

Both groups of diseased buffaloes examined in the present study exhibited common signs including altered appetite, scanty feces, mild abdominal distension, dehydration, rumen hypomotility or stasis and systemic disturbances. These clinical features are in agreement with previous reports in calves (Sardari 2007) and adult cattle (Fubini and Ducharme 2016) with omental bursitis. Splashing sound by abdominal ballottement was an interesting sign that indicates the presence of gases with fluid in omentum or the retention of ingesta and gases in intestinal loop secondary to the paralytic ileus with peritonitis. Expiratory grunting sound was an additional sign appearing in few cases of both conditions and was expressed as a sign of severe abdominal pain. As recorded previously (Abdelaal et al. 2009) it is obvious that buffaloes express clinical signs of pain with difficulty. Melena that indicates presence of acid hematin in feces was observed in 6 cases of OB and 4 cases of LP. The same sign was previously recorded in buffaloes with hemorrhagic abomasal ulcer (Abdelaal et al. 2017). This manifestation is an indicator for the presence of bleeding resulting from abomasal ulcer that tinged with HCL in abomasum forming acid hematin. Loss of consciousness was bad prognostic sign and was clear in all cases of LP.

In the current study, ultrasonography was an ideal tool for description of the site, size, and character of the lesion. The lesions of OB and LP were restricted at the area of ventral abdomen and that confirmed the involvement of the abomasum. The anechoic to hypoechoic appearance of the lesions depend on the degree of suppuration and duration of the lesions that could reflect an idea about the prognosis. The fluid of OB was characterized by echogenic stippling indicating microbial gas production by microbes. In general, the abomasal ulcer is referred to ulceration of the inner mucosa of the abomasum which starts by slight abrasion (type 1). It is then extended to include the sub-mucosa with its blood vessels (type 2). Finally, the ulcer extends to involve the whole layers of the abomasal wall (type 3 perforated stage), whereas the perforation is then complicated by LP and OB (type 4). This result was previously described in cattle with OB secondary to type 4 abomasal ulcer (Braun 2016). The reticulum of affected buffaloes was normal in shape and character with the exception of hypomotility. The hypomotility could result from the effect of endotoxemia and indigestion.

Besides aimed at diagnosis and treatment follow-up, ultrasonography was also used as a guide to the aspiration of lesional fluid. The aspiration with lavage as well as other treatment strategies provided a good step for treatment of OB cases while they were non-effective in LP cases. This result was previously monitored in another report of internal abscesses in buffaloes (Abdelaal et al. 2017).

Significant increase in the total proteins in both OB and LP might be attributed to the inflammation and associated hyperglobulinaemia (McPherson and Pincus 2017). Hypoalbuminemia which appeared in OB might be due to the switching of the synthesized protein in the liver, during the inflammation, towards increasing the synthesis of the positive acute phase proteins which is concomitant with a decrease in the albumin production. These acute phase proteins have a major role in the prevention of the inflammation and help in the healing process (Cray et al. 2009).

The increase in the activity of pepsinogen was observed in both the buffaloes with OB and LP. Pepsinogen is considered the inactive form of pepsin. It is the important enzyme responsible for proteolysis of the gastric juice. Increasing the activation of pepsinogen, by enhancement the acidity of gastric contents, into pepsin can cause ulcers in animals. There are two main findings responsible for increasing the activity of serum pepsinogen. The first one involves increased vascular and epithelial permeability allowing the leaking of pepsinogen into the blood and the second one involves the hyper-secretion of pepsinogen directly into the blood stream, in a retrograde direction, from zymogenic cells (Mesarič 2005). Our finding demonstrates that increasing the pepsinogen concentration reflects the damage in the abomasal mucosa.

Sodium has the largest concentration of ions in the extracellular fluid thereby predominantly responsible for maintaining osmotic pressure. Both OB and LP may have water retention due to the compensating renal responses, leading to normal level of the serum sodium. The chloride level was decreased in association with the inflammatory process in the OB and LP cases. The amounts of potassium or chloride might not have been lost from the animal’s body, but the observed decrease in their concentrations might be due to the acid-based imbalance or alteration. Some of the most common causes for the decrease in the serum chloride level is the peritoneal inflammation (Dezfouli et al. 2012). The decrease in the level of chloride without a decrease in the proportion of the sodium may accompanied by the appearance of metabolic alkalosis associated with vagus indigestion, abomasal torsion and abomasal ulcer (Smith 2014). The concentration of potassium in serum does not always reflect the balance of potassium but it is affected by factors that alter the internal balance as well as those that change the external balance. The depletion in the body’s potassium store or its redistribution from the ECF space into the ICF predispose to hypokalemia (Smith 2014). In our study, the hypokalemia might be due to the inflammation which is responsible for anorexia or might be due to the potassium loss from the digestive system.

It could be concluded that the biochemical alterations in buffaloes with OB and LP could provide some information in regard to the differentiation of these cases but still it is not considered a confirmatory tool for the disease differentiation. However, ultrasonography provides a strong useful diagnostic tool and guidance for the detection and differentiation between both diseases. The OB did respond to treatment with good prognosis, whereas LP has a poor prognosis. Therefore, the quick and accurate diagnosis could help veterinarians for achieving appropriate decision making to combat such difficult disease.
